# The Poor-Law Infirmary

**Published:** 1907-07-20

**Authors:** 


### Poor-law Department.

### THE POOR-LAW INFIRMARY.

### SOME ESSENTIALS OF ECONOMICAL WORKING.

Control from Within.

When a company of practical people set about to establish a club, a hotel, or a business of any kind their first step is to appoint a manager. Aware that the success of the undertaking will depend entirely upon the extent to which the manager can be brought to identify his interests with their own, they offer him an adequate salary and stimulate him by every means in their power to rule the establishment with a single aim. To this end they give him a large discretion in the choice of the persons who are to work under him and carry out his orders; and although they may prefer to keep the ultimate right of dismissal, as of engagement, in their own hands, they refuse no reasonable requests to this effect which may be brought forward by one on whom their confidence reposes. Having taken every precaution to secure the right person for the post as manager, they recognise, in fact, that he is in a better position than themselves to form a judgment about the internal affairs of the establishment he is called upon to administer, and they refrain, as a matter of principle, from interference with details. These principles of government, familiar to all business men, are so obviously just that it is surprising to find that in municipal matters in the control of Poor-law institutions they are violated in every particular.

Under existing conditions in local government no one has any real authority, not even the guardians themselves. The number of important things about which the guardians have no power to give orders is one of the first hard lessons which an enthusiastic person elected to this ambiguous office is compelled to learn. The clerk is the nearest approach to an authority which the system affords. Yet he is hampered and kept under in every conceivable manner. His work demands incessant attendance in his office, at a superfluity of meetings. The process of getting authority to carry out his simplest duties would suffice to execute the necessary work forty times over, and he must teach a succession of well-meaning but ignorant superior officers the elements of his duties before he can get leave to perform them. He has no power to dismiss so much as a drunken porter, taken in the act, since all his subordinates are independent of his jurisdiction, although they may be nominally subject to his orders. The steward on whom the responsiblity of dealing with many thousand pounds worth of goods depends is at an even greater disadvantage as regards authority, and the common practice of raising to this position a junior clerk, and continuing to pay him the salary of a clerk, though it may have a spurious appearance of economy, is an exceedingly extravagant proceeding in the long run.

The crying need of the workhouse or Poor-law infirmary is a house-keeper. The extent to which Poor-law infirmaries are understaffed as regards nurses makes it utterly impossible that the matron can add to her already over-heavy tasks that of domestic supervision. The work should be undertaken by an official able to devote his entire time to the duties of supervision and control. There is no reason why the post should not be held by a woman, but the heavy nature of the duties and the number of men-servants whose work needs controlling are indications that a male steward is more suitable. It should be made a good appointment, since it demands high qualities, and not least among them that power of making authority felt, which is in itself of high market value. In voluntary institutions personal devotion to the work and to the particular institution is a factor in securing and retaining disinterested service. But rate-supported institutions can hardly expect to evoke this sentiment of personal enthusiasm, and hence they must attain their object of attracting the right kind of official by offering a living wage. Such a steward or manager, appointed after successful work in some similar position of authority, would act as confidential adviser to the guardians in all the transactions over which they now so often fall into grievous difficulties. If building or alterations are to be undertaken, the manager should supervise the plans, advise upon the expenditure, and watch the execution of the work with the same care that an ordinary householder gives to his affairs in similar case. When contracts are to be entered upon, the manager should sort and sift the samples sent in, and prepare such information for the use of the guardians as may place them in a position to make a wise decision. He must scrutinise on its delivery every article supplied to the institution, and personally superintend the reception, storage, and distribution of the goods. He should be directly responsible for the quality of the catering right through the establishment. The servants are also engaged and dismissed according to his recommendation, and the cook, as well as all other servants, is to work immediately under his direction and according to time-tables made out in his office. He is responsible for the economical use of every article supplied to the patients and staff; and he makes it his business to know the exact amount required and to check every week the consumption per head of each article. He should be responsible for the cleanliness of the whole establishment, except that of the wards, and for the control of such special departments as the laundry and bakery.

Week by week he should account for the heavy responsibilities resting upon him by preparing for the guardians returns showing the cost of provisions for patients and staff, the quantity of coal and gas consumed in the various departments, the wages-sheet, and such other particulars as may be necessary to check the expenditure in the households under his charge.

